# SGLT2i: beyond the glucose-lowering effect

**DOI:** 10.1186/s12933-020-01071-y

**Published:** 2020-06-26

**Authors:** Lihua Ni, Cheng Yuan, Guopeng Chen, Changjiang Zhang, Xiaoyan Wu

**Affiliations:** 1 Department of Nephrology, Zhongnan Hospital of Wuhan University, 169 Donghu Road, Wuhan, Hubei 430071 China; 2 Department of Gynecological Oncology, Zhongnan Hospital, Wuhan University, Wuhan, 430071 People’s Republic of China; 3 Institute of Model Animal, Wuhan University, Wuhan, 430071 China; 4 School of Basic Medical Sciences, Wuhan University, Wuhan, 430071 China; 5 Department of Cardiology, Renmin Hospital of Wuhan University, Zhang Road No. 99, Wuhan, Hubei 430060 China; 6 Cardiovascular Research Institute, Wuhan University, Wuhan, 430060 People’s Republic of China; 7 Hubei Key Laboratory of Cardiology, Wuhan, 430060 People’s Republic of China; 8 Cardiovascular Disease Center, Enshi Central Hospital, Enshi, 445000 People’s Republic of China

**Keywords:** Sodium-glucose cotransporter-2 inhibitors (SGLT2i), Type 2 diabetes, Cardiovascular disease, Renal disease

## Abstract

Sodium/glucose cotransporter-2 inhibitors (SGLT2i) are a new type of glucose-lowering drug that can reduce blood glucose by inhibiting its reabsorption in proximal tubules and by promoting urinary glucose excretion. SGLT2i are widely used in the clinical treatment of type 2 diabetes mellitus (T2DM). In recent studies, SGLT2i were found to not only reduce blood glucose but also protect the heart and kidney, which can significantly reduce cardiovascular events, delay the progression of renal failure, greatly improve the quality of life of patients, and reduce medical expenses for families and society. As adverse cardiac and renal events are the most common and serious complications of T2DM, it is very important to understand the cardio- and renoprotective mechanisms of SGLT2i. This article reviews the historical development, pharmacological mechanism, heart and kidney protection and safety of SGLT2i. The information presented provides a theoretical basis for the clinical prevention and treatment of diabetes and its complications and for the development of new glucose-lowering drugs.

## Background

Type 2 diabetes (T2DM) is a metabolic disease that is commonly associated with obesity, dyslipidemia, hypertension, heart failure, hyperuricemia, renal failure and hyperuricemia and affects individuals worldwide [1–3]. In addition, patients with T2DM have an increased risk of cardiovascular or renal complications, which are leading causes of morbidity and mortality [4, 5]. Although several oral and intravenous glucose-control drugs have been widely used, concerns about cardiorenal complications in these populations still attract considerable attention [6]. Therefore, a more comprehensive approach to cardiovascular and renal risk management in T2DM has emerged. Sodium/glucose cotransporter-2 inhibitors (SGLT2i) block SGLT2 located in the early proximal renal tubule, which leads to increased urinary glucose excretion and subsequently decreased serum glucose concentrations [7, 8]. These glucose-lowering effects are independent of insulin action. To date, SGLT2i has been demonstrated to reduce major adverse cardiovascular events and hospitalization for heart failure [9, 10]. In addition, these drugs showed surprising effects on the progression of renal complications, such as lowering serum creatinine, reducing albuminuria, and decreasing death due to renal disease [11, 12]. Interestingly, these cardiorenal protective effects appear to be independent of glucose-control efficacy. Considering these encouraging findings, SGLT2i are highly recommended for patients with T2DM with high cardiovascular or renal risks. Therefore, the mechanisms that drive the cardiorenal protection of SGLT2i should be elucidated. This review aims to provide an update on the extraglycemic effects of SGLT2i that may contribute to cardiorenal protection.

## The discovery and development of SGLT2i

In 1835, French chemists isolated a natural substance called phloridzin from the root bark of apple trees, which was first used to treat infectious malaria [13]. Further study found that the chemical can hinder the reabsorption of glucose in the kidney, increase glucose excretion in urine, reduce blood sugar levels and improve insulin resistance [13, 14]. However, phloridzin is a kind of nonselective SGLTi (it inhibits both SGLT1 and SGLT2), which can be hydrolyzed by β-glucosidase in the small intestine with low bioavailability (only 15%), and SGLT1i are prone to cause diarrhea, dehydration and other adverse reactions [15]. Therefore, the research and development of drugs with high selectivity, high bioavailability and safety have become an urgent problem to be solved. In recent years, with the development of medical science and technology, scientists have chemically modified the structure of phloridzin and gradually developed many new derivatives, including O-glucoside, C-glucoside, N-glucoside and nonglucoside, and named these derivatives SGLT2i. C-glucosides have been widely used in the clinic because of their high SGLT2 selectivity and safety. At present, there are four kinds of SGLT2i approved by the US Food and Drug Administration (empagliflozin, canagliflozin, dapagliflozin, and ertugliflozin), as shown in Table 1. In March 2017, dapagliflozin became the first SGLT2i approved for use in China. Additionally, sotagliflozin, the first dual SGLT2/1 inhibitor, improves glycated hemoglobin in adults with diabetes, with beneficial effects on body weight and blood pressure [16].

**Table 1:** Brief introduction to the features of SGLT2i

Trade name	①Empagliflozin ②Canagliflozin ③Dapagliflozin ④Ertugliflozin
Target organ	Proximal tubule of kidney
Favorable aspects	①Lower blood glucose
	②Lower blood pressure (reduce systolic BP by 2.46 mmHg and diastolic BP by 1.46 mmHg)
	③Lower body weight (3 kg–4 kg reduction)
	④Lower glycosylated hemoglobin (0.7%–1% reduction)
	⑤Increase high-density lipoprotein (HDL) cholesterol, and decrease triglycerides.
	⑥Reduce albuminuria
	⑦Reduce uric acid (10%–15% reduction)
	⑧Increase hematocrit (2–4% increase)
Unfavorable aspects	①Urinary tract infection
	②Genital fungal infection
	③Ketoacidosis
	④Fracture
	⑤Malignant tumor
	⑥Hypotension
	⑦Dehydration
	⑧Hypoglycemia
	⑨Lower limb amputation

*BP* blood pressure

## Expression and pharmacological action of SGLT2i

In healthy people, approximately 160 g–180 g glucose is filtered from the glomerulus and reabsorbed into the blood circulation every day, so healthy people have almost no urinary sugar; in people with diabetes, the concentration of glucose undergoing glomerular filtration is increased significantly and can reach 180 g–240 g. This level is much higher than the renal glucose threshold, leading to an increase in urinary sugar. The entry of glucose into eukaryotic cells requires two membrane-associated transporters: SGLT (SGLT1 and SGLT2) and glucose transporter (GLUT) [17]. SGLT transports glucose against the concentration gradient by active transport, while GLUT transports glucose along the concentration gradient in a way that facilitates diffusion. Familial renal glucosuria (FRG), a rare disease, is characterized as persistent glucosuria despite normal concentrations of serum glucose [18, 19]. In general, FRG has been considered a benign condition that does not require any specific treatment. Mutations in the *SGLT2* gene have recently been confirmed as responsible for most FRGs [20–22], leading to isolated glucosuria. Therefore, the study of SGLT has become a research target for glucose-lowering therapy.

There are 6 subtypes of SGLTs [19], and their main distribution and functions are shown in Table 2. The two most important members, SGLT1 and SGLT2, are related to glucose transport and are the focus of current research. SGLT1 is mainly expressed in the small intestine and renal proximal tubule (S3 segment) and is a high-affinity, low-volume membrane transporter, while almost all SGLT2, which is a low-affinity, high-volume membrane transporter, is expressed in the S1 segment of the renal proximal tubule [23]. Ninety percent of glucose reabsorption is performed by SGLT2, and the remaining 10% is performed by SGLT1 [23]. SGLT1 has multiple organ targets but does not target the kidney; thus, further study of highly selective SGLT2i is of great significance.

**Table 2:** Distribution and function of various SGLTs

Protein	Major sites of expression	Function
SGLT1	Small intestine	Transmembrane transport of glucose and galactose through sodium-glucose cotransport proteins in the brush margin of the small intestine and proximal convoluted tubules of the kidney
	Trachea	
	Heart	
	Proximal tubule of the kidney (segment S3)	
SGLT2	Proximal tubules of kidney (segment S1 and S2)	Cotransport of sodium and glucose in the S1 segment of the renal proximal convoluted tubule
SGLT3	Small intestine	Transport of sodium (not transport of glucose)
	Lungs	
	Uterus	
	Thyroid	
	Testicles	
SGLT4	Small intestine	Transport of glucose and mannose
	Kidneys	
	Liver	
	Lungs	
	Stomach	
SGLT5	Renal cortex	Unknown
SGLT6	Spinal cord	Transport of inositol and glucose
	Brain	
	Small intestine	
	Kidney	

The aglycones of SGLT2i can competitively bind to glucose transporters, effectively inhibiting the activity of SGLT2 in renal proximal convoluted tubules, reducing glucose reabsorption by renal tubular epithelial cells, promoting urinary glucose excretion and exerting glucose-lowering effects. SGLT2i can also inhibit sodium reabsorption in renal tubules, increasing urinary sodium concentration in the distal end of the proximal convoluted tubule and proximal end of the distal tubule, improving renal tubuloglomerular feedback, reducing renal hyperfiltration, and reducing sodium and glucose toxicity in the proximal tubule [24]. Interestingly, SGLT2i do not rely on insulin or islet β-cells in vivo to exert pharmacological effects. These inhibitors can not only reduce blood glucose and glycosylated hemoglobin but also improve the function of islet β-cells and reduce insulin levels in the body [25]. However, Bonner et al. [26] demonstrated that SGLT2 is expressed in glucagon-secreting α cells of the pancreatic islets; SGLT2i treatment by dapagliflozin promotes glucagon secretion and hepatic gluconeogenesis in healthy mice, limiting the decrease in serum glucose induced by fasting. They believed that SGLT2 was an endocrine regulator. This opinion was further supported by Sargent et al. [27]. Therefore, SGLT2i is also promising for patients with insulin resistance and islet insufficiency.

## Protective effect of SGLT2i on the cardiovascular system

Cardiovascular events are one of the most common and serious complications in patients with diabetes. Epidemiological studies in the UK have found that the mortality rate of cardiovascular events in patients with diabetes is 3.25-fold higher than that in nondiabetic patients [28]. Chinese data show that 30% of diabetic patients also have cardiovascular events [29], and these events account for 20% of diabetes mortality in China [30]. Therefore, it is very important to actively prevent and treat the cardiovascular complications of diabetes.

Many clinical studies have found that SGLT2i have a protective effect on the cardiovascular system and can significantly reduce the risk of cardiovascular events. The results of the high-profile EMPA-REG OUTCOMES (Empagliflozin Cardiovascular Outcome Event Trial in Type 2 Diabetes Mellitus Patients) study, which included 7020 patients with T2DM complicated with cardiovascular diseases who were followed up for 192 weeks, were published in 2015 [31]. The study showed that the number of MACE (major adverse cardiovascular events, including cardiovascular-related death, nonfatal cerebral infarction, and nonfatal myocardial infarction) decreased by 14%, the number of cardiovascular-related deaths decreased by 38%, the number of hospitalized cases of heart failure decreased by 35%, and the mortality rate decreased by 32% in the empagliflozin treatment group compared with the placebo group. A follow-up study also found that empagliflozin showed a cardiovascular protective effect from the first month to the third month of treatment, and with the extension of treatment time, the cardiovascular protective effect was still significantly different from that of the placebo group. Later, the Canagliflozin Cardiovascular Assessment Study (CANVAS) program [32] and the Dapagliflozin effect on Cardiovascular Events-Thrombolysis in Myocardial Infarction 58 (DECLARE-TIMI58) trial [33] showed similar trends (reduction in cardiovascular death and hospitalization for heart failure).

Interestingly, the recently published DAPA-HF trial [34] demonstrated that dapagliflozin significantly reduces cardiovascular death and hospitalization for heart failure in patients with heart failure with reduced ejection fraction. More importantly, these benefits were observed both in the presence or absence of diabetes and were consistent in relation to background heart failure therapy [35]. Sezai et al. pointed out that canagliflozin was also effective in patients with diabetes and chronic heart failure [36]. Petrie et al. observed that dapagliflozin could reduce the risk of worsening heart failure and cardiovascular events in patients with heart failure independently of diabetes status [37]. However, no change in cardiac function parameters was estimated by impedance cardiography in diabetes patients who received dapagliflozin versus placebo for 12 weeks [38], which may have been related to the small sample size, short duration treatment, and limited correlation of impedance cardiography parameters with the gold standard examination of cardiac function.

The basic characteristics of the main clinical studies of SGLT2i regarding the cardiovascular system are summarized in Table 3. At present, SGLT2i have been included in the first-level prevention guidelines of the 2019ACC/AHA for cardiovascular disease, and metformin combined with dapagliflozin is recommended as the preferred treatment regimen for glucose-lowering drug treatment of diabetes [39].

**Table 3:** Basic characteristics of the main clinical studies of SGLT2i in the cardiovascular system

	EMPA-REG OUTCOME [31]	CANVAS Program [32]	DECLARE-TIMI58 [33]	DAPA-HF Trial [34]
Inclusion criteria	Type 2 diabetes and high cardiovascular risk	Inadequately controlled type 2 diabetes with a history or high risk of CV events	Type 2 diabetes, CV risk factors and moderately impaired renal function	Chronic heart failure, left ventricular ejection fraction ≤ 0.40% and elevated NT-proBNP
Total number (n)	7020	10,142	16,170	4744
Primary outcomes	CV death, nonfatal MI and nonfatal stroke	CV death, nonfatal MI and nonfatal stroke	CV death, nonfatal MI, nonfatal stroke, or hospitalization for HF	CV death or hospitalization for HF or an urgent heart failure clinic visit

*CV* cardiovascular, *MI* myocardial infarction, *NT-proBNP* N-terminal of the prohormone brain natriuretic peptide, *HF* heart failure

The protective mechanism of SGLT2i on the cardiovascular system involves many aspects, including direct and indirect effects on the heart, which are summarized as follows (Table 4) [40–45].

**Table 4:** Protective effects of SGLT2i on the cardiovascular system

Action	Mechanism
Direct effects	Inhibit myocardial Na^+^/H^+^ exchange
	Improve myocardial metabolism
	Reduce cardiac preload
	Reduce afterload
	Reduce cardiomyocyte apoptosis and improve myocardial fibrosis
	Reduce the synthesis of adipokines, cytokines and epicardial adipose tissue
	Attenuate sympathetic nerve activity
Indirect effects	Improve blood glucose
	Promote weight loss
	Lower blood pressure
	Reduce proteinuria, delaying the progression of renal disease

Direct effects: (1) SGLT2i inhibit myocardial Na^+^/H^+^ exchange (NHE). By inhibiting cardiac Na^+^/H^+^ exchange activity, SGLT2i increase the concentration of sodium ions in mitochondria and reverse electrolyte disorders in patients with heart failure. (2) SGLT2i improve myocardial metabolism. These inhibitors can improve myocardial energy metabolism, increase myocardial oxygen supply, promote ATP energy storage, increase oxygen uptake and transformation at the mitochondrial level, increase ketone bodies, lower the insulin-to-glucagon ratio, inhibit myocardial fibrosis, switch from glucose to ketone utilization during myocardial metabolism and reverse myocardial remodeling. (3) SGLT2i reduce cardiac preload. They can reduce cardiac preload and myocardial oxygen consumption by osmotic diuresis. More importantly, osmotic diuresis induced by SGLT2i, a diuretic mechanism that is distinctly different from that of other diuretic classes, leads to greater electrolyte-free water clearance and subsequently greater fluid clearance from the interstitial fluid space than from the circulation, resulting in congestion relief with minimal impact on blood volume, arterial filling, and organ perfusion [46]. (4) SGLT2i reduce afterload. They can lower blood pressure by osmotic diuresis and increase urinary sodium excretion, improve cardiovascular function by reducing oxidative stress and endothelial cell inflammation, and then reduce cardiac afterload. (5) SGLT2i reduce cardiomyocyte apoptosis and improve myocardial fibrosis. SGLT2i leads to progression or inhibition of apoptosis [47]. SGLT2i could attenuate cardiac fibrosis by alleviating oxidative stress and TGF-β production and regulating macrophage polarization [48, 49]. Interestingly, several studies have demonstrated the relationship between NHE and cardiomyocyte apoptosis [50] and myocardial fibrosis [51, 52]. Active NHE-1 (a predominant isoform of NHE in cardiomyocytes) leads to the accumulation of intracellular sodium, further increasing intracellular Ca^2+^, which triggers myocardial apoptosis in addition to necrosis [50]. A possible link between NHE-1 activity and fibrosis may be based on the fact that NHE-1 is a downstream effector of several fibrosis-related signaling systems [51]. (6) SGLT2i reduce the synthesis of adipokines, cytokines and epicardial adipose tissue. The alteration of adipokine production and/or action has been proposed as a common mechanism of cardiovascular disease [53]. Ectopic fat deposition in the form of epicardial fat could lead to the genesis of heart failure [54]. SGLT2i could restore the balance between pro- and anti-inflammatory adipokines. (7) SGLT2i attenuate sympathetic nerve activity [55]. Activation of the sympathetic nervous system is closely related to the onset and progression of heart failure, and the mechanism by which SGLT2i suppress sympathetic nerve activity is not yet fully understood. One possible explanation is described by mediating ketone body metabolism.

Indirect effects: (1) SGLT2i improve blood glucose. Studies have confirmed that hyperglycosylated hemoglobin and hypoglycemia events are risk factors for cardiovascular events, but SGLT2i can reduce glycosylated hemoglobin and reduce the risk of hypoglycemia, resulting in cardiovascular benefits. (2) SGLT2i promote weight loss. Obesity is an independent risk factor for cardiovascular disease. SGLT2i cause glycosuria and negative energy balance, thereby leading to body weight loss [56]. SGLT2i can reduce the occurrence of cardiovascular events by promoting weight loss. (3) SGLT2i lower blood pressure [57]. It is well known that hypertension is a common complication of diabetes and one of the risk factors for cardiovascular disease. SGLT2i reduce systolic BP by 2.46 mmHg and diastolic BP by 1.46 mmHg, while they reduce 24-h ambulatory systolic and diastolic BP by 3.76 mmHg and 1.83 mmHg, respectively [58, 59]. SGLT2i can exert antihypertensive effects in many ways (such as decreased uric acid levels, metabolic fuel switching (ketogenic) activity, reduced body weight, hemodynamic mechanisms secondary to volume depletion caused by diuresis and natriuresis, and so on [57]) and thus play a role in cardiovascular protection. (4) SGLT2i reduce proteinuria, delaying the progression of renal disease. Proteinuria and renal insufficiency are risk factors for cardiovascular events in patients with diabetes. SGLT2i can reduce proteinuria by reducing glomerular hyperfiltration. In addition, SGLT2i also have a good renal protective effect (described in more detail below), delaying the progressive damage of diabetic nephropathy.

The beneficial effects of SGLT2i in preventing cardiovascular events have been widely recognized in the clinic. We anticipate that further insight into the underlying mechanisms will be needed in the future.

### Protective effect of SGLT2i on the kidney

Diabetic nephropathy is a leading cause of end-stage kidney disease in China, specifically ranking second in China. Worldwide, the incidence of diabetic microalbuminuria is 39%, and the annual incidence of albuminuria is 3.1% [60]. In China, 30% of T1DM and 20% of T2DM patients eventually progress to diabetic nephropathy [61]. Diabetic nephropathy is one of the most serious chronic complications in patients with diabetes, and it is also the main cause of death. Therefore, it is of great significance to actively prevent and treat diabetic renal complications.

According to a large clinical trial by Wanner et al. [62], compared with the placebo control group, the empagliflozin treatment group showed a significantly reduced incidence of kidney disease, risk of kidney deterioration, and risk of renal replacement therapy. The relative risk of doubling the ratio of urinary albumin to creatinine was also significantly reduced. The 2019 ADA guidelines [63] recommend the use of SGLT2i (class C), which have been shown to reduce the risk of kidney progression and cardiovascular events, in patients with T2DM complicated with chronic kidney disease. In 2019, the Chinese Clinical Guide for the Prevention and Treatment of Diabetic Renal Disease clearly stated [64] that SGLT2i have renoprotective effects in addition to hypoglycemic effects. SGLT2i (grade A) are preferred when patients with diabetic nephropathy cannot control hyperglycemia with metformin.

The possible mechanisms by which SGLT2i protect the kidney, including the direct and indirect effects on the kidney, are summarized as follows (Table 5) [23, 65, 66].

**Table 5:** Protective effects of SGLT2i on the kidney

Action	Mechanism
Direct effects	Improve glomerular hyperfiltration
	Reduce renal oxygen consumption
	Reduce renal inflammatory reactions
	Restore the mode of cellular energy metabolism
Indirect effects	Improve blood glucose
	Improve blood pressure
	Decrease uric acid levels
	Promote weight loss
	Increase the level of glucagon
	Reduce the level of insulin
	Promote diuresis

Direct effects: (1) SGLT2i improve glomerular hyperfiltration. Under physiological conditions, renal tubule-glomerular feedback can regulate the tension of entering glomerular arterioles and maintain the stability of renal function; however, under the condition of hyperglycemia, the reabsorption of SGLT2-mediated sodium and glucose in renal proximal tubules increased, the mechanism of tubule feedback was damaged, the tension of entering glomerular arterioles was abnormal, renal blood perfusion increased, vascular wall pressure increased, the basement membrane thickened, and glomeruli were injured. SGLT2i can block the reabsorption of sodium and glucose in the proximal tubules, regulate renal tubule-glomerular feedback and reduce glomerular ultrafiltration. (2) SGLT2i reduce renal oxygen consumption. SGLT2i can inhibit the active reabsorption of sodium in proximal tubules, thus reducing renal energy consumption and protecting the kidney. (3) SGLT2i reduce renal inflammatory reactions. Under high glucose conditions, the expression of proinflammatory factors, growth factors, profibrotic mediators, advanced glycation end products and reactive oxygen species in the proximal tubules of the kidney increased, and the renal cortex thickened. SGLT2i can inhibit the expression of inflammatory factors and reduce the infiltration of inflammatory factors to reduce renal inflammation and delay changes in structure and function and the progression of fibrosis in the process of diabetic nephropathy. (4) SGLT2i restore the mode of cellular energy metabolism. They can improve renal energy metabolism and increase oxygen uptake and transformation at the level of mitochondria.

Indirect effects: (1) SGLT2i improve blood glucose. Hyperglycemia causes glucotoxic damage to the kidney. SGLT2i lower blood glucose and reduce the renal hypertrophy, injury and inflammation caused by glucotoxicity. (2) SGLT2i improve blood pressure. When the body is in a state of hypertension for a long time, the self-regulating ability of renal vessels decreases, leading to renal dysfunction and proteinuria. SGLT2i can slightly lower blood pressure and indirectly affect renal function. In addition, a previous study proved that improved morning home systolic BP with dapagliflozin was independently related to alleviation in albuminuria in patients with diabetic nephropathy [67]. Additionally, SGLT2i may reduce body weight and blood pressure in nondiabetic patients [68]. (3) SGLT2i decrease uric acid levels. High levels of uric acid can form crystals and deposit in the kidney, reducing the glomerular filtration rate. In addition, other studies have confirmed that serum uric acid may promote the occurrence and development of diabetic nephropathy by mediating endothelial dysfunction, RAAS overactivation and the inflammatory response. SGLT2i promote osmotic diuresis and uric acid excretion, thus reducing the burden on the kidney. (4) SGLT2i promote weight loss. On the one hand, obesity results in mechanical pressure on the kidney, causing renal hypoxia; on the other hand, obesity affects renal hemodynamics (including increased renal blood flow, glomerular hyperfiltration and renal tubule sodium retention) and increases glomerular filtration rate and glomerular volume. SGLT2i can reduce abdominal and peripheral fat and body weight through glycosuria-related calorie loss and osmotic diuresis, thus improving renal hypoxia and hemodynamics and protecting the kidney. (5) SGLT2i increase the level of glucagon. Glucagon can dilate blood vessels and increase renal blood flow, renal filtration and electrolyte excretion, thereby maintaining renal function. SGLT2i can protect the kidney by lowering blood glucose and increasing glucagon. (6) SGLT2i reduce the level of insulin. Insulin can promote the proliferation of renal cells and the extracellular matrix and damage renal function. SGLT2i can reduce the level of blood glucose, reduce insulin secretion and decrease the burden on the kidney. (7) SGLT2i promote diuresis. The synergistic effect of SGLT2i and proximal tubule Na^+^/H^+^ can produce diuresis and blood pressure reduction, decreasing the burden of the kidney.

It should be emphasized that SGLT2i can significantly delay the progression of renal failure but cannot treat diabetic nephropathy. Some studies have found that SGLT2i can also cause a transient decline in renal function and return to normal within a few weeks [69, 70]. Clinical attention should be paid to this issue.

### Safety evaluation of SGLT2i

SGLT2i have a unique glucose-lowering effect, which plays a protective role in many organs, but adverse reactions also need to be highlighted. The more common complications include adverse reactions related to glucose-lowering therapy and urinary glucose (such as genitourinary infection and ketoacidosis) and off-target adverse reactions (including fracture, lower limb amputation risk and tumor).

Statistically, the incidence of reproductive tract infection, generally mild-to-moderate infection, after treatment with SGLT2i is 4.8% [71]. Due to differences in anatomical structure, the incidence rate of females is higher than that of males. The mechanism through which SGLT2i increase the incidence of reproductive tract infection may be by promoting glucose excretion through urine, increasing the concentration of glucose in the genitourinary tract, and increasing the risk of bacterial and fungal infection. Therefore, attention should be paid to genitourinary tract hygiene when using SGLT2i. For genitourinary tract infection or recurrent infection within half a year, SGLT2i should be used cautiously or not recommended. However, some major outcome trials suggest that SGLT2i are not associated with an increased risk of urinary tract infections (UTIs) [31–33]. Wilding et al. suggested that it might be the disease that increases the chances of infection, not the drug, and that the benefits of the SGLT2i seem to outweigh these risks of infection [72].

The incidence of ketoacidosis caused by SGLT2i is relatively rare, approximately 0.1% [31]. The mechanism may be related to the effect of SGLT2i on the levels of insulin and glucagon in the body, the promotion of fat decomposition and the β-oxidation of fatty acids, and the increase in ketone body production in the liver [73]. It should be noted that the number of cases of diabetic ketoacidosis has been increasing, especially the euglycemic manifestation associated with SGLT2i treatment [73]. Many have postulated possible mechanisms for SGLT2i-associated diabetic ketoacidosis [74, 75], which make diabetic ketoacidosis a legitimate, small risk associated with SGLT2i administration. Therefore, during use, we need to pay attention to the serum ketone body levels and pH values.

The CANVAS study pointed out that canagliflozin can increase the rate of bone metastasis, reduce the bone mineral density of the hip, and increase the risk of fracture [32]. The mechanism underlying the increased risk of fracture may be related to the increased serum phosphate level, decreased vitamin D level, weight loss and so on [76]. Therefore, for people at high risk of fracture, SGLT2i need to be used in a cautious manner.

An approximately twofold increase in the risk of lower limb amputation associated with SGLT2i compared with placebo was observed in clinical trials [32, 33, 77]. However, the recent CREDENCE trial reported no significant difference in lower limb amputation risk between the canagliflozin and placebo groups [78]. Meta-analysis of 14 RCTs (N > 26,000) demonstrated that SGLT2i as a class was not significantly associated with amputation risk, but subgroup analysis showed an increased risk for canagliflozin compared with other oral antihyperglycemic agents. All patients with diabetes have a higher risk of lower limb infections, ulcers and amputation [79]. However, it remains unclear whether SGLT2i increase this risk, suggesting that we need to evaluate patient foot health on a regular basis.

A previous study [80, 81] indicated that canagliflozin increased the risk of developing renal tubule tumors, pheochromocytomas and testicular Leydig cell tumors in rats. The mechanism may be related to the inhibition of intestinal carbohydrate absorption, the increase in calcium excretion in renal tubules and the synthesis of luteinizing hormone induced by canagliflozin. However, there are differences between animals and people, and animal models cannot completely replace clinical research. Further clinical trials showed that the risk of bladder cancer and breast cancer in the dapagliflozin treatment group was higher than that in the control group [82]. However, the study did not conduct tumor screening and could not determine whether the tumor was caused by dapagliflozin treatment. Therefore, there is still controversy about whether SGLT2i can induce tumorigenesis.

Large-sample multicenter clinical trials and additional basic experimental verification are still needed. Additional adverse reactions reported clinically include hypotension, dehydration, hypoglycemia and so on [83]. However, the risk of hypoglycemia is low unless coadministered with insulin and insulin secretagogues (e.g., sulfonylureas, glinides). Thus, consideration needs to be given to reducing the dose of either agent used in combination with an SGLT2i. Mechanistically, SGLT2i are not associated with increased hypoglycemia risk [84].

The above adverse reactions suggest that medical staff should prescribe drugs in a reasonable manner, with an understanding of the complications to properly avoid risks. At the same time, the research and development of safe glucose-lowering drugs with high selectivity is also a difficult problem that we urgently need to overcome.

## Conclusion and prospect

The drug treatment model of diabetes is facing profound changes. SGLT2i are a new type of glucose-lowering drug that, because of their unique action mechanism, do not depend on insulin or islet β-cells in vivo to exert pharmacological effects. Not only can they reduce blood glucose and glycosylated hemoglobin, but they can also improve the function of islet β-cells and reduce the level of insulin in the body. Further study has gradually revealed additional roles of these inhibitors other than reducing blood sugar. At present, SGLT2i have shown good cardiorenal protective effects, but there are also some adverse reactions. Only by understanding and paying attention to the pharmacological mechanism can we understand these limitations, use such drugs reasonably and take precautions to avoid risks. To date, SGLT2i have shown bright prospects and are expected to become clinical first-line drugs for T2DM. Further study on the mechanism is still needed in the future. In addition to improving the therapeutic effect, it is urgent to reduce adverse drug reactions, including possible systemic and carcinogenic risks.

## Data Availability

Not applicable.

## Abbreviations

SGLT2i: Sodium/glucose cotransporter-2-inhibitors
T2DM: Type 2 diabetes mellitus
GLUT: Glucose transporter
